# The Prognostic Value of Inflammatory Markers in Paediatric Acute Kidney Injury

**DOI:** 10.3390/jcm15031099

**Published:** 2026-01-30

**Authors:** Flavia Chisavu, Lazar Chisavu, Mihai Gafencu, Diana Hanu, Ruxandra Maria Steflea, Teofana Otilia Bizerea-Moga, Ramona Stroescu

**Affiliations:** 1 4th Pediatric Clinic, “Louis Turcanu” Children’s Clinical and Emergency Hospital, Iosif Nemoianu 2, 300011 Timisoara, Romania; farkas.flavia@umft.ro (F.C.); mgafencu@umft.ro (M.G.); diana.hanu@umft.ro (D.H.); steflea.ruxandra@umft.ro (R.M.S.); stroescu.ramona@umft.ro (R.S.); 2 Centre for Molecular Research in Nephrology and Vascular Disease, Faculty of Medicine “Victor Babes”, “Victor Babes” University of Medicine and Pharmacy Timisoara, 300041 Timisoara, Romania; 3 Discipline of Nephrology, University of Medicine and Pharmacy “Victor Babes”, Eftimie Murgu Square No. 2, 300041 Timisoara, Romania; 4 Nephrology Clinic, Emergency Clinical County Hospital “Pius Brinzeu”, 300723 Timisoara, Romania; 5 Department XI of Pediatrics-3rd Pediatric Discipline, “Victor Babes” University of Medicine and Pharmacy Timisoara, Eftimie Murgu Sq. No. 2, 300041 Timisoara, Romania; 6 Department of Doctoral Studies, “Victor Babes” University of Medicine and Pharmacy Timisoara, Eftimie Murgu Square No. 2, 300041 Timisoara, Romania; 7 Department XI of Pediatrics-1st Pediatric Discipline, Center for Research on Growth and Developmental Disorders in Children, “Victor Babes” University of Medicine and Pharmacy Timisoara, Eftimie Murgu Sq. No. 2, 300041 Timisoara, Romania; bizerea.teofana@umft.ro

**Keywords:** children, acute kidney injury, mortality, interleukin 6, inflammatory markers

## Abstract

**Background/Objectives**: Acute kidney injury (AKI) is common in children. Several inflammatory markers proved their utility in predicting AKI, especially in adults, but their utility in children’s populations is still under debate. **Methods**: We performed an observational retrospective cohort study on children admitted to the “Louis Turcanu” Emergency County Hospital for Children from Timisoara, Romania. We evaluated the utility of procalcitonin, C reactive protein, lactate dehydrogenase, ferritin, interleukin 6, albumins and erythrocyte sedimentation rate in predicting AKI and mortality in children. **Results**: The final cohort consisted of 131 children. The incidence of AKI was 39.6%, with more than half (61.1%) admitted in the intensive care unit. Out of twelve deaths, 11 were encountered in the AKI group. Patients from the AKI group presented higher levels of several inflammatory markers: lactate dehydrogenase, C reactive protein, procalcitonin, LDH, CRP, PCT and ferritin and lower albumins. Only ferritin, C reactive protein and procalcitonin could predict AKI development. Procalcitonin seems to increase mortality, but in the adjusted regression model, only AKI increased mortality. AKI increased mortality by 4.11 times (95%CI = 1.07–15.66, *p* = 0.038). **Conclusions**: Procalcitonin, C reactive protein, and ferritin proved to be predictors of AKI, yet none of the inflammatory markers influenced mortality. AKI is still an important independent mortality factor regardless of the underlying disease spectrum.

## 1. Introduction

Acute kidney injury (AKI) is common in children [1,2], with a diverse range of aetiologies that share different degrees of inflammatory responses, which further perpetuates the decline of kidney function [3]. The role of certain inflammatory markers such as C reactive protein (CRP), serum albumin, interleukin 6 (IL-6), lactate dehydrogenase (LDH), ferritin levels [3,4,5], erythrocyte sedimentation rate (ESR) [6], and procalcitonin (PCT) [7] proved to be positively linked to AKI severity, besides the usefulness of these biomarkers in predicting AKI development. Although extensive research has examined these inflammatory biomarkers in adults, there are relatively few studies involving children, which are focused on target groups [8,9,10,11].

Current evidence traces the influence of early exposure to inflammation over the risk of chronic inflammation throughout the life span [12,13]. Also, given that over 50% of the worldwide population deaths are attributable to inflammation-related diseases such as autoimmune diseases, ischemic heart diseases, stroke, neoplasia, diabetes mellitus, neurodegenerative conditions and chronic kidney disease [14], the role of certain factors have been linked to a dysregulated immune response. Depending on the extent of the inflammation, whether it is systemic or local, and the degree of inflammation—high grade or low-grade, short-term duration or persistent, non-resolving inflammatory states—the outcomes could drift from healing, trigger removal and tissue repair to collateral damage leading to a silent and continuous activation of distinct immune components from those engaged during an acute event [15].

Despite current evidence, the role of IL-6 is not entirely clear in children with AKI. While the role of IL-6 is linked to the severity of the underlying disease spectrum [11], in states of decreased renal function, cytokines have a lower clearance, especially in states of inflammatory diseases, thus theoretically leading to higher IL-6 levels. IL-6 is the major regulator of acute phase protein synthesis in inflammatory states [16]. IL-6 synthesis is associated with the first stage of inflammation as IL-6 moves from the bloodstream to the liver and initiates rapid active phase proteins such as CRP, fibrinogen, serum amyloid A, and haptoglobin while it reduces the production of albumin, transferrin, and fibronectin [16]. CRP, produced by the liver in response to systemic inflammation, is a non-specific acute-phase protein that increases in both infectious and non-infectious inflammatory disorders [17]. Superior to CRP is PCT, a specific inflammatory biomarker that can distinguish bacterial infections from inflammatory states [18]. However, it is important to emphasize the utility of these markers in clinical practice as all inflammatory biomarkers have different timing and kinetics [19]. IL-6 levels rapid rise, often preceding PCT or CRP elevations, offers an excellent overall view over tissue damage or severe inflammation [19].

Il-6 correlates with the onset and severity of AKI, as well as many other inflammatory markers such as PCT, CRP, ESR, LDH, and ferritin [4,20,21,22,23]. Although these inflammatory biomarkers can predict AKI, it is crucial to acknowledge that higher levels can be associated with other inflammatory conditions, and also, the ideal thresholds values and optimal timeframe can vary among different patient groups. The development of AKI is associated with high morbidity and mortality along with a prolonged hospital stay [2,23]. Yet, the heterogeneous data about inflammatory biomarkers that could predict AKI length or severity are under consideration; especially in specific paediatric fields, their utility is still under debate for the general population. The objective of this study is to examine if and which specific inflammatory markers are associated with the risk of AKI development and severity. Given the variable spectrum of children’s diseases, often presenting with an acute disorder or an acute event over a chronic condition, attention should focus on the utility of inflammatory biomarkers for end organ damage such as AKI, and to evaluate the systemic response with specific of non-specific inflammatory markers. This study aimed to fill the knowledge gap by examining the role of inflammatory biomarkers in forecasting AKI development in a large mixed paediatric population.

## 2. Materials and Methods

### 2.1. Ethical Considerations

The research followed the guidelines outlined in the Declaration of Helsinki. Approval for the study was granted by the Hospital’s Medical Ethics Committee (20607/6 December 2024).

### 2.2. Study Design and Participants

We performed a single centre, retrospective, observational cohort study of all hospitalised patients between 1 August 2014 and 31 March 2024 admitted to “Louis Turcanu” Emergency County Hospital for Children in Timisoara, Romania. All patients under 18 years old were screened for at least one serum IL-6 determination and at least two serum creatinine (Scr) levels during hospitalization. In addition, we included only patients that developed acute kidney injury at least after 24 h of admission. Initially, we identified 207 patients with IL-6 measured in the first 24 h after admission. We excluded 76 patients from the initial cohort due to the lack of at least two serum creatinine measurements within 7 days, in order to be able to identify the occurrence of AKI. Patients were assessed for several other markers of inflammation, including lactate dehydrogenase (LDH), procalcitonin (PCT), C-reactive protein (CRP), erythrocyte sedimentation rate (ESR), ferritin, and levels of serum albumin. The final cohort included 131 children. If a patient was admitted to the hospital more than once, only their first hospitalization was considered.

### 2.3. Data Collection and Definition

Data on baseline characteristics—including gender, environment, age, and demographics—as well as biological information were collected from both the Hospital Information System and the Laboratory Information System. The measurement of IL-6 levels was conducted at an external partner laboratory utilizing the electrochemiluminescence technique. AKI was defined as an increase in Scr of 26.5 µmol/L within 48 h or to more than 1.5 times baseline known or presumed to have occurred within 7 days or the nadir Scr in 7 days from admission, according to the 2012 KDIGO Clinical Practice Guidelines [24]. AKI staging was considered as an increase of more or equal to 26.5 µmol/L or increase to more than or equal to 150–200% (1.5- to twofold) from baseline in stage 1, more than 200–300% increase from baseline in stage 2 and  >300% increase from baseline, or more than  ≥354 µmol/L or the initiation of renal replacement therapy (RRT) in stage 3 AKI. All patients with documented anuria for over 12 h were considered as having stage 3 AKI. Serum creatinine was measured using the Abbott Jaffe method with plasma paediatric creatinine reference intervals based on age.

Data was analysed by two different operators at the same time to eliminate potential bias.

The collected data are from the first day of hospitalization. Individuals who had incomplete data were left out of the statistical analysis for that particular parameter. We did not replace the missing data. The missing data are as follows: 0.7% for C reactive protein, 9.9% for procalcitonin, 15.2% for albumins, 9.1% for leucocytes, 38.1% for erythrocyte sedimentation rate, 38.1% for ferritin and 26.7% for lactate dehydrogenase.

### 2.4. Statistical Analysis

The continuous variables were tested for normality using Shapiro–Wilk test. Normally distributed ones are expressed as mean and standard deviation and non-normally ones as median and interquartile range. Percentages are used to express non-continuous variables, which are then analyzed using the Chi-square test. We used either *t*-test or ANOVA to evaluate normally distributed variables and Mann–Whitney or Kruskall–Wallis tests for non-normally distributed ones. We present the risk of death as odds ratio and 95% confidence interval. We used a Kaplan–Meier curve to present the risk of death stratified by the development of AKI. To evaluate the prediction value of the inflammatory biomarkers, we performed several logistic regressions with each inflammatory marker as the independent variable and AKI occurrence as the dependent one. In addition, we present the ROC-AUC curves for these biomarkers. We evaluated the impact of inflammatory markers on mortality using logistic regression models with mortality as dependant variable and each inflammatory marker as independent one. We adjusted these logistic regression models only for AKI occurrence. All the data were analysed using MedCalc^®^ Statistical Software version 23.4.5 (MedCalc Software Ltd., Ostend, Belgium; https://www.medcalc.org; 2025) (accessed on 28 December 2025).

## 3. Results

Over the 10-year study period, we found 131 patients with at least one IL-6 level measured during hospital stay and at least two serum creatinine measurements. Patients were divided into two groups: those who experienced AKI and those who did not (non-AKI).

The baseline characteristics are presented in Table 1.

The incidence of AKI was 39.6%. Patients who developed AKI more often came from the rural area, presented longer hospital stay and higher ICU admission rates with longer ICU stay. Out of twelve deaths, eleven were met in the AKI group.

Further, we analysed the biological data (measured during the first day of admission) as presented in Table 2.

The analysis revealed that out of the inflammatory markers, ESR and IL-6 levels did not reach a statistically significant difference between groups. However, patients from the AKI group presented higher levels of several inflammatory markers: LDH, CRP, PCT, and ferritin levels, and lower serum albumin. Also, they had lower haematological parameters with higher liver enzymes as opposed to those from the non-AKI group. The baseline serum creatinine did not differ between the two groups, only the peak level of creatinine reached during hospitalization.

Further, we compared the median values of the biomarkers in our cohort stratified by the AKI severity. Out of the inflammatory markers, only LDH levels in stages 2 and 3 of AKI (*p* < 0.0001), PCT in stages 2 and 3 (*p* < 0.0001), and ferritin stages 1 and 2 (*p* = 0.002) reached statistical significance when compared to those without AKI—Table 3.

In the subgroup analysis of patients with and without sepsis, stratified by AKI development, albumins were lower in the AKI group, while PCT and LDH were higher—regardless the presence of sepsis—while ferritin was higher in AKI only in patients without sepsis, as shown Supplemental Table S1.

The patients were stratified by the admission causes—Table 4. The cardiac causes included cardiac malformations (Fallot tetralogy, atrial septal defect, ventricular septal defect), pericarditis, myocarditis, and cardiac arrhythmias. The renal causes were related to infection such as acute pyelonephritis with or without congenital renal malformation. Neurological implication was due to intracerebral haemorrhage, obstructive hydrocephaly, meningitis. Inflammatory causes were represented by autoimmune diseases (juvenile idiopathic arthritis, lupus erythematosus, paediatric inflammatory multisystem syndrome (PIMS), Kawasaki syndrome). Haematological diseases included both diseases from the oncological spectrum, mostly solid tumours (neuroblastoma, hepatoblastoma, cerebral tumour), and haematological cancers (leukaemia lymphoma, granulomatosis). Also, combined immunodeficiencies, aplastic anaemia, idiopathic thrombocytopenic purpura was included in the analysis. Surgical causes were represented by acute appendicitis, peritonitis, gastroschisis, varicocele, inguinal hernia, hydrocele, cholecystitis, diaphragmatic hernia, intestinal malrotation, intestinal occlusion, and oesophagus atresia. Pulmonary causes were upper and lower respiratory tract infections, empyema, abscess, pleurisy, tuberculosis.

Based on the cause of admission we evaluated the mean values of the inflammatory markers to underline the inflammatory response on different disease spectrum as seen in Table 5.

The analysis revealed renal disorders were more likely to reach higher levels of both IL-6 and PCT levels, while ferritin was linked to acute phase reactants in haemato-oncological, neurological and inflammatory diseases. CRP was increased in cases of infection regardless of the affected organ. LDH was higher in almost all cases, with emphasis on neurological and pulmonary disorders. Albumins were more likely to be lower in surgical patients and neurological ones. ESR presented no statistical difference among studied subgroups.

**Figure 1:**
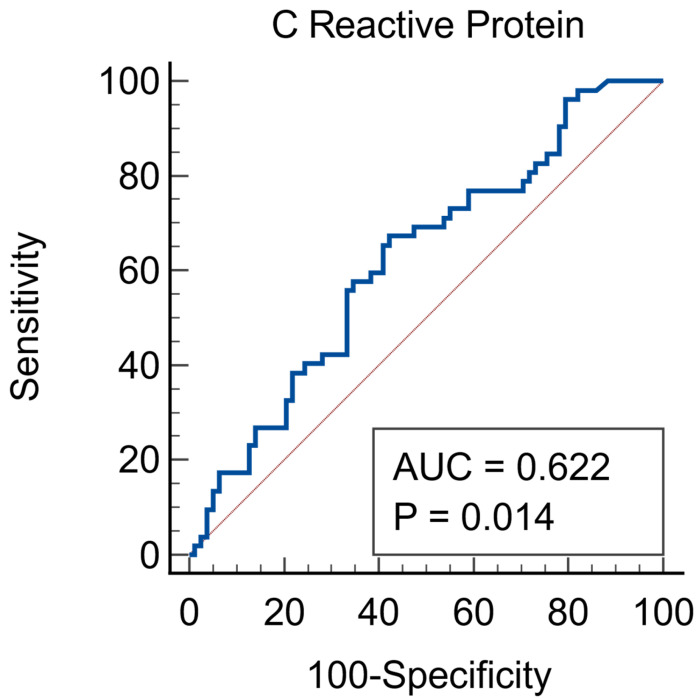
C reactive protein as a predictor of AKI. Red line = diagonal line, blue line = ROC curve.

Given the heterogeneity of these different inflammatory markers, we further evaluated the prediction of AKI using several ROC curve analyses. Firstly, IL-6 was not a predictor of AKI. CRP was a fair predictor of AKI, with an ROC AUC of 0.622 (95%CI = 0.5333–0.706, *p* = 0.014), associated criterion of more than 45.97 mg/dL, sensitivity of 67.31% and specificity of 57.96%, as seen in Figure 1.

**Figure 2:**
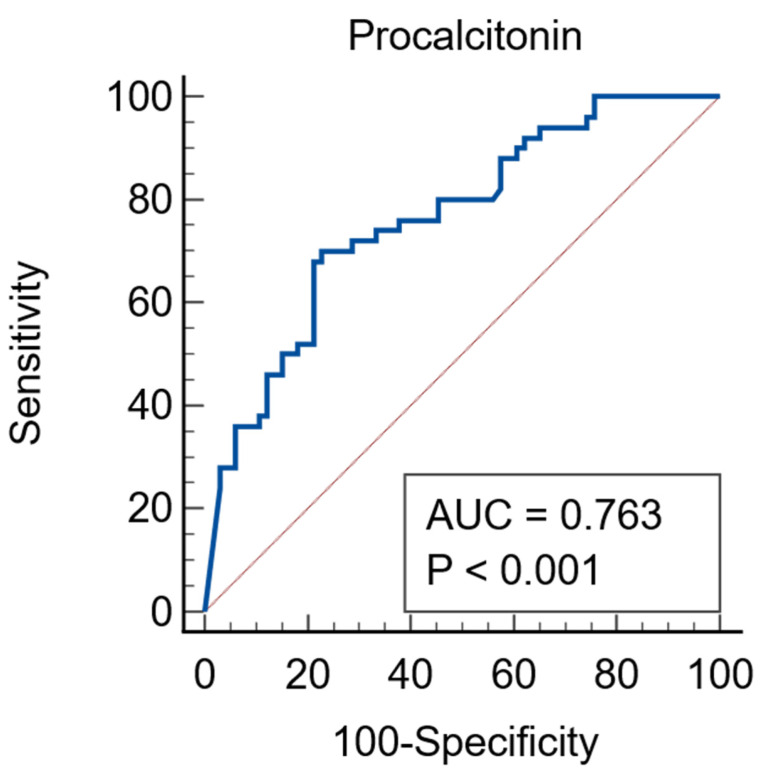
Procalcitonin as a predictor of AKI. Red line = diagonal line, blue line = ROC curve.

Procalcitonin was a good predictor of AKI, the ROC curve having an AUC of 0.763 (95%CI = 0.676–0.837), *p* < 0.0001, associated criterion of >4.07 ng/mL, sensitivity of 70% and specificity of 77.27%—Figure 2.

**Figure 3:**
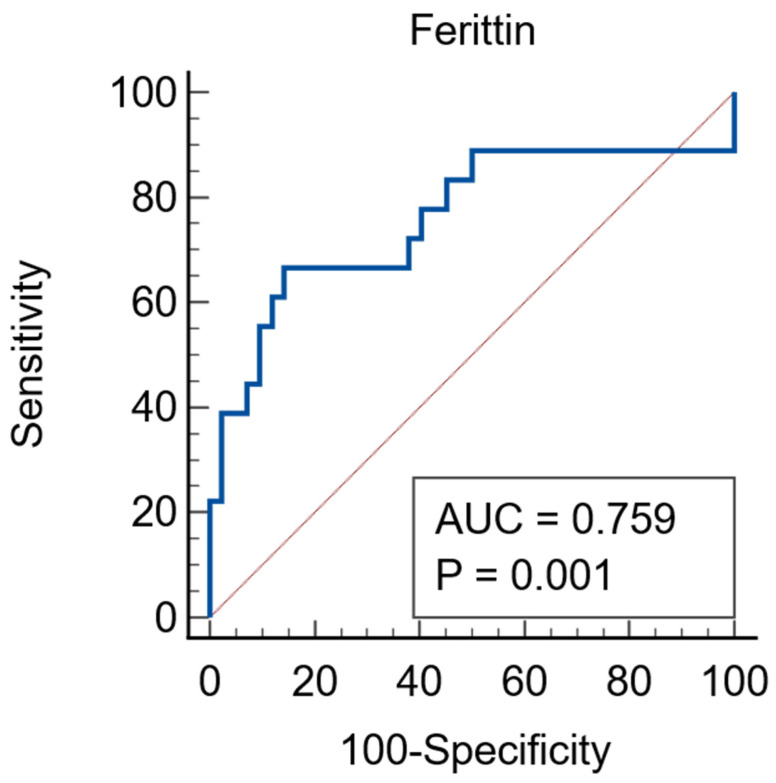
Ferritin as a predictor of AKI. Red line = diagonal line, blue line = ROC curve.

Ferritin was a good predictor of AKI, the ROC curve having an AUC of 0.759 (95%CI = 0.631–0.86), *p* = 0.0013, associated criterion of >705 ng/mL, sensitivity of 66.67% and specificity of 85.71%—Figure 3.

**Figure 4:**
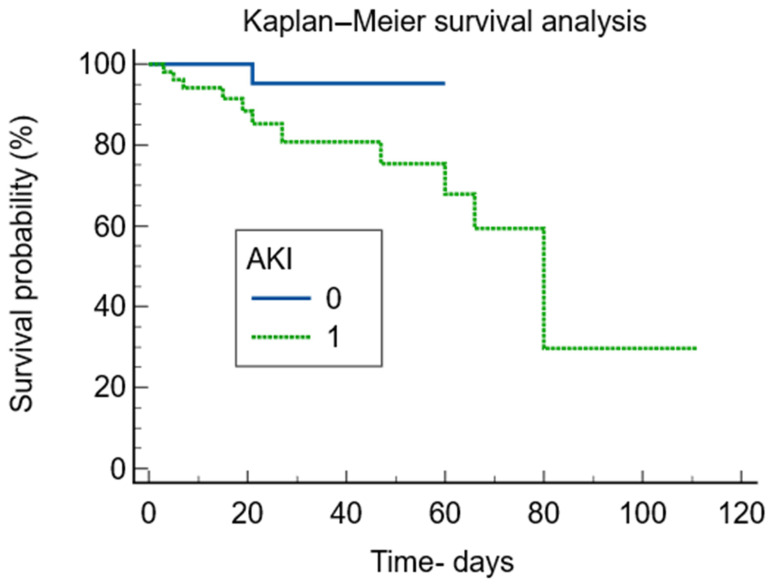
Kaplan–Meier survival analysis.

The Kaplan–Meier survival analysis stratified by AKI and non-AKI was statistically significant, with a *p*-value of 0.038, and the presence of AKI increased the risk of death by 4.11 times (95%CI = 1.07–15.66)—Figure 4.

We evaluated the impact on mortality of the inflammatory markers using the logistic regression model, and only procalcitonin influenced mortality, each unit increase in procalcitonin generating a 1.8% higher mortality risk—(95%CI = 1.003–1.032, *p* = 0.0147), AUC = 0.748 (95%CI = 0.659—0.824). When we adjusted the regression for AKI, PCT was not an independent mortality predictor (*p* = 0.27).

## 4. Discussion

Our unicentric retrospective observational study of children with various diseases screened for inflammatory markers found that only PCT, CRP, and ferritin were able to predict the development of AKI. However, the interaction between these biomarkers and AKI could signal a cause-and-effect relationship, and they can also state a non-causal effect. For instance, ferritin was higher in patients that developed AKI only in the presence of sepsis, while LDH and PCT were higher in the AKI group regardless the presence of sepsis. Patients with AKI had worse biological parameters, longer hospital stay, higher ICU admission and higher mortality rates. Out of the inflammatory markers, only PCT seems to increase mortality. However, in the adjusted regression, PCT no longer influenced mortality and only AKI remained an independent mortality risk factor. These results could reflect how inflammation intervenes in AKI development in children. Clearly, the underlying disease is a key factor for the dynamic and variability of inflammatory markers.

The serum inflammatory markers such as IL-6, PCT, CRP, LDH, ESR, ferritin and albumin had been extensively studied in adults and only in a few specific paediatric populations, with a distinct role in AKI prediction and prognosis [4,6,11,25,26,27,28]. Despite the consecrated role of IL-6 in AKI prediction and prognosis [11,29,30], our study failed to prove a link between AKI prediction or severity in a large mixed cohort study with both consecrated inflammatory diseases and severe non-inflammatory diseases associated with or without markers of inflammation, as described in the causes of admission. IL-6 has a pleiotropic activity in multiple biological states with dual function [3,31,32,33,34,35,36]. Our study found that IL-6 levels were generally higher in cases of kidney disorders and lung diseases, which is consistent with previous research [27]. Infection markers such as PCT and CRP were elevated in renal disorders with or without AKI, in parallel with the highest IL-6 levels, underlying the importance of the acute setting of the disease with recovery, especially in states of infection. On the other hand, patients with haemato-oncology diseases had higher levels of IL-6, with lower PCT and CRP levels, which emphasizes the dysregulation of the pro and anti-inflammatory role of cytokines in these patients, regardless of acquired infections [37,38]. Nevertheless, patients with inflammatory disorders have higher inflammatory markers, with variable levels consistent with the literature [39]. Yet, despite these results, the role of inflammatory markers in clinical practice reflects the need for AKI screening, as patients with worse biological parameters at admission and younger age are at a higher risk of AKI development, regardless of baseline serum creatinine. We found that our high rate of AKI, 4 out of 10 admitted children, was much higher compared with the latest meta-analysis of worldwide AKI incidence in hospitalized children [2], with high ICU admission rates and overall longer hospital stay, consistent with previous studies [2,40,41,42]. High vigilance is needed in patients with sepsis as they have a higher risk of developing AKI. PCT, a well-established marker of inflammation, and CRP levels were both elevated in patients with infections. Both biomarkers proved to be good predictors of AKI development. These results are consistent with previous studies which reported higher CRP, PCT and IL-6 to be associated with AKI [4,26].

Serum ferritin, a nonspecific known inflammatory marker, besides being an iron status marker [43], possess significant early predictive AKI value in children [28] with positive mortality correlation [44]. Similar outcomes were observed in our research, primarily due to systemic inflammation present in renal, neurological, haematological-oncological, and autoimmune disorders. However, despite being an independent AKI predictor, we could not show an increased risk of mortality. Overall, the only mortality risk factor in our cohort was represented by AKI. In the first analysis, higher levels of PCT seemed to increase the mortality risk; however, in the adjusted regression, PCT no longer reached statistical significance. Similar results were found in critically ill patients, where serum PCT levels were significantly higher in patients with AKI but were not effective in predicting mortality [7].

Nevertheless, AKI proved to be an independent mortality risk factor in both children and adults [1,2,23,45], as it did in our study. Mortality was overall low, as we are considering a mixed paediatric population with various causes of hospitalization. Yet, patients with AKI had twenty times more deaths compared with the ones without AKI, revealing the importance of kidney injury as an independent risk factor in children.

The main limitation of our study is the retrospective nature with a reduced number of patients. Due to the relatively small number of patients, our results should be carefully interpreted, and we consider that larger studies with a prospective nature are needed to validate our results. On the other hand, the high percentage of patients admitted to ICU could limit the generality of these results in non-critical patients. Nevertheless, the interpretation of the predictive value of inflammatory markers should remain cautious, given the retrospective design and the heterogeneity of the studied population. The strength of our study includes the heterogenous causes of admission, from upper respiratory tract infection to autoimmune diseases or haemato-oncological ones, from all paediatric age groups. Also, the various inflammatory markers included are another strength, including IL-6.

## 5. Conclusions

In conclusion, the role of inflammatory markers in predicting paediatric AKI is set up by the underlying disease. Despite the pleiotropic nature of IL-6, its role in paediatric AKI was not proved in this study. PCT, CRP, and ferritin proved to be predictors of AKI, yet none of the inflammatory markers influenced mortality. AKI is still an important independent mortality factor regardless of the underlying disease spectrum.

## Figures and Tables

**Table 1:** Baseline characteristics.

Parameter	Total N = 131	AKI N = 52	Non-AKI N = 79	*p* Value
Age years *	5.1 (1.42–10.87)	4.5 (0.25–10)	6 (2.27–11.02)	0.053
Sex—males	74 (56.5%)	30 (57.7%)	44 (55.7%)	0.822
Urban environment	72 (55%)	22 (42.3%)	50 (63.3%)	0.018
Deaths	12 (9.2%)	11 (21.2%)	1 (1.3%)	0.0001
ICU Admission	80 (61.1%)	41 (78.8%)	39 (49.4%)	0.0007
ICU stay—days (only for ICU patients) *	8 (5–18)	15 (6–20)	6 (3–13)	0.0034
Sepsis	42 (32.1%)	22 (42.3%)	20 (25.3%)	0.0423
Hospital stay—days *	14 (8–29)	21.5 (13–48.5)	10 (6–22)	<0.0001

Legend: N = number; AKI = acute kidney injury; ICU = intensive care unit; * = median and interquartile. Statistical tests: Chi-square test or Mann–Whitney test.

**Table 2:** Biological data.

Parameter	Total N = 131	AKI N = 52	Non-AKI N = 79	*p* Value
LDH * IU/L	307 (250–554)	543 (293–1140)	276 (209–326)	<0.0001
Peak serum creatinine * umol/L	44 (28.25–72.25)	86 (54.5–119)	32 (23.75–44)	<0.0001
Baseline creatinine * umol/L	27 (21–36)	28.5 (18–34.5)	27 (22–37.75)	0.91
Urea * mmol/L	4.74 (3.45–8.48)	8.56 (5.55–19.3)	3.93 (2.87–5.15)	<0.0001
GOT * IU/L	35 (21.25–72)	61.5 (29.5–163.5)	29 (19–42.25)	<0.0001
GPT * IU/L	23 (13–45)	34 (15–102.5)	20 (12–35.25)	0.0026
Hemoglobin ^1^ g/dL	10.6 (2.14)	9.91 (2.25)	11.04 (1.96)	0.0029
Thrombocytes * N/mmc	242,000 (129,250–378,500)	150,000 (56,000–266,500)	293,000 (182,000–436,250)	<0.0001
CRP * mg/L	55 (12.6–177.68)	98.81 (26.82–236.51)	37.03 (10.25–142.23)	0.0184
Procalcitonin * ng/mL	3.05 (0.21–18.23)	11.31 (1.37–83.63)	0.81 (0.09–3.91)	<0.0001
IL-6 * pg/mL	34.15 (10.5–115.45)	47.02 (14.24–121.4)	28.3 (6.96–104.97)	0.145
Albumins ^1^ g/L	28.12 (5)	25.5 (4.86)	30.15 (4.12)	<0.0001
Leucocytes * N × 1000/mmc	11.45 (7.06–18.17)	13.33 (5.05–20.59)	10.9 (7.06–17.31)	0.284
ESR * mm/h	44.5 (18–81)	45 (14–85)	40 (18–74.75)	0.771
Ferritin * ng/mL	230 (79.5–848)	1218 (226–2336)	147 (67–651)	0.0016

Legend: N = number; AKI = acute kidney injury; LDH = lactate dehydrogenase; GOT = glutamic oxaloacetic transaminase; GPT = glutamic pyruvic transaminase; CRP = C reactive protein; IL-6 = interleukin 6; ESR = erythrocyte sedimentation rate; mg = milligrams, L = liter, umol = microimole, mmol = millimole, IU = international units, mL = milliliter, g = grams, dL = deciliter, mmc = cube millimeter, ng = nanograms, pg = picograms, mm = millimeter, h = hour. * = median and interquartile; ^1^ = mean and standard deviation. Statistical tests: *t*-test or Mann–Whitney test.

**Table 3:** Inflammatory markers stratified by AKI severity.

Parameter	Without AKI	AKI Stage 1	AKI Stage 2	AKI Stage 3	*p* Value
IL-6 * pg/mL	28.3 (6.96–104.97)	43.59 (15.41–325.87)	16.89 (8.57–75.09)	62.62 (32.5–189.8)	0.079
CRP * mg/L	37.03 (10.25–142.23)	74.97 (9.37–302.3)	64.68 (23.9–177.7)	129.9 (51.57–240.88)	0.072
Ferritin * ng/mL	147 (67–651)	1344 (162.25–6132)	1602 (971–2292)	184 (46.75–597.25)	0.002 ^a^
LDH * IU/L	276 (209.75–326.5)	276.5 (264–808)	569 (411.5–722.25)	675 (319.5–1445)	0.00001 ^b^
Albumins ** g/L	30.15 (4.11)	28.28 (6.6)	26.44 (3.78)	23.74 (3.99)	<0.001 ^c^
ESR * mm/h	40 (18–74.75)	57 (16.25–113)	32.5 (10–54)	60 (29.5–95)	0.49
PCT * ng/mL	0.81 (0.09–3.91)	4.93 (0.22–22.31)	4.12 (0.44–17.38)	35.73 (7.26–100)	<0.00001 ^d^

Legend: AKI = acute kidney injury; LDH = lactate dehydrogenase; CRP = C reactive protein; IL-6 = interleukin 6; ESR = erythrocyte sedimentation rate; mg = milligrams, L = liter, IU = international units, mL = milliliter, g = grams, ng = nanograms, pg = picograms, mm = millimeter, h = hour. * = median and interquartile; ** = mean and standard deviation. ^a^ = stage 0 vs. stages 1 and 2; ^b^ = stages 2 and 3 vs. stages 0 and 1; ^c^ = stage 0 vs. stages 2 and 3; ^d^ = stage 3 vs. stages 0, 1 and 2, stage 2 vs. stage 0. Statistical tests: ANOVA and Kruskal–Wallis test.

**Table 4:** Causes of admission.

Parameter	Total	AKI	Non-AKI	*p* Value
Cardiac	13 (9.9%)	2 (3.8%)	11 (13.9%)	0.06
Renal	8 (6.1%)	5 (9.6%)	3 (3.8%)	0.175
Neurological	10 (7.6%)	6 (11.5%)	4 (5.1%)	0.17
Inflammatory	25 (19.1%)	7 (13.5%)	18 (22.8%)	0.18
Hematological	17 (13%)	9 (17.3%)	8 (10.1%)	023
Surgical	16 (12.2%)	6 (11.5%)	10 (12.7%)	0.84
Pulmonary	31 (23.7%)	9 (17.3%)	22 (27.8%)	0.16
Others	11 (8.4%)	8 (15.4%)	3 (3.8%)	0.026

Legend: AKI = acute kidney injury.

**Table 5:** Causes of admission and mean inflammatory markers.

Parameter *	IL-6 pg/mL ^1^	PCT ng/mL ^2^	Ferritin ng/mL ^3^	CRP mg/L ^4^	LDH IU/L ^5^	Albumins g/L ^6^
Cardiac	28.3 (7.67–105.37)	0.61 (0.09–1.97)	120 (53–252)	20.56 (1.9–90.67)	299 (233.5–370.25)	30.47 (4.37)
Renal	99.98 (9.74–637.55)	100 (18.99–100)	71 (35–1769)	119.83 (21.18–175.69)	229 (201.25–928)	26 (4.39)
Neurological	39.05 (13.96–147.3)	38.91 (10.14–100)	17,105 (5254–19,898)	222.3 (4.07–247.67)	928 (497–1229)	24.91 (5.95)
Inflammatory	12.7 (4.74–49.16)	0.81 (0.14–2.79)	396 (78.25–775.5)	61.43 (24.027–183.78)	270 (204.5–324)	29.75 (5.07)
Haematological	48.76 (22.98–249.85)	0.3 (0.09–1.9)	1517 (170.75–2816.75)	31.72 (8.49–124.5)	341.5 (271–459)	27.94 (4.93)
Surgical	25.53 (12.7–49.27)	1.05 (0.07–5.48)	-	68.45 (0.3–104.34)	310 (268–341.5)	24.62 (4.46)
Pulmonary	76.63 (23.34–163.82)	4.36 (0.97–19.99)	194 (54–391)	52.06 (29.89–260.37)	493.5 (273.5–890)	29.45 (4.39)
Others	20.43 (5.59–200.6)	5.42 (3.46–28.8)	157 (115.5–216)	130.35 (15.5–183.11)	300 (215–521)	26.16 (4.44)
*p* value	0.045 *	0.0001 *	0.041 *	0.243 *	0.015 *	0.011 **

Legend, only albumins are expressed as mean and standard deviation, all other inflammatory markers are expressed as median and interquartile range. LDH = lactate dehydrogenase; CRP = C reactive protein; IL-6 = interleukin 6; ESR = erythrocyte sedimentation rate; mg = milligrams, L = liter, IU = international units, mL = milliliter, g = grams, dL = deciliter, ng = nanograms, pg = picograms, ^1^ inflammatory vs. renal, hematological and pulmonary; ^2^ others versus cardiac, inflammatory, hematological and surgical; cardiac versus renal, neurological and pulmonary; renal versus inflammatory, hematological and surgical; neurological versus inflammatory, hematological, surgical; inflammatory vs. pulmonary; hematological vs. pulmonary; surgical vs. pulmonary. M + IQR = median and interquartile range; ^3^ neurological vs. all other causes; cardiac vs. hematological; ^4^ no statistical differences; ^5^ neurological vs. all categories except pulmonary; inflammatory vs. hematological and pulmonary; ^6^ cardiac vs. others, neurological and surgical; neurological vs. inflammatory and pulmonary; inflammatory vs. surgical. * Kruskal–Wallis test, ** ANOVA test.

## Data Availability

All the data used is present in the article. The access to the database will be granted only after the request to the corresponding author at e-mail chisavu.lazar@umft.ro and after the approval of all the authors.

## Supplementary Materials

The following supporting information can be downloaded at https://www.mdpi.com/article/10.3390/jcm15031099/s1, Table S1: Inflammatory markers values stratified by AKI and sepsis.

## Abbreviations

The following abbreviations are used in this manuscript:

AKI	Acute kidney injury
IL-6	Interleukin 6
PCT	Procalcitonin
CRP	C reactive protein
