# Supplementary material for: The Prognostic Value of Inflammatory Markers in Paediatric Acute Kidney Injury
Source: J Clin Med. 2026 Jan 30;15(3):1099. doi: 10.3390/jcm15031099 (PMC12898657; doi:10.3390/jcm15031099)
Supplement: Supplementary file 1 [file jcm-15-01099-s001.zip › jcm-4106900-supplementary.pdf]

Supplemental Table S1. inflammatory markers values stratified by AKI and sepsis.

	Sepsis			Without sepsis		
	AKI	Without AKI	P value	AKI	Without AKI	P value
IL-6 pg/ml, M+IQR	47.97 (18-118.9)	78.42 (23.43-246.3)	0.334	47.02 (10.39-123.9)	20.1 (3.88-86.13)	0.085
CRP mg/l, M+IQR	143.79 (74.97-234.1)	191.77 (45.01-276.87)	0.989	44.79 (7.2-238.9)	25.51 (2.07-79.28)	0.082
Ferritin ng/ml, M+IQR	223.5 (145-2336)	280 (127.5-525)	0.914	1324 (772.5-4511)	135 (57-651)	0.0006
LDH mg/dl, M+IQR	551 (254-1687)	250 (190-281)	0.011	543 (302-820)	280 (214-327)	<0.0001
Albumins g/l, mean and SD	25.03 (5.16)	29.01 (4.51)	0.019	25.82 (4.68)	30.55 (3.94)	<0.0001
ESR mm/h, M+IQR	63.5 (24-97.5)	32.5 (10-85)	0.198	39 (8-72)	45 (20-72.5)	0.395
PCT ng/ml, M+IQR	30.06 (8.45-100)	3.91 (1.18-24.77)	0.011	4.78 (0.78-27.14)	0.21 (0.07-1.26)	<0.0001

Legend: AKI=acute kidney injury; LDH=lactate dehydrogenase; CRP=C reactive protein; IL-6=interleukin 6; ESR=erythrocyte sedimentation rate; mg=milligrams, l=litre, umol=millimoles, ml=millilitre, g=grams, dl=decilitre, ng=nanograms, pg=picograms, g=grams, mm=millimetre, h=hour. M+IQR=median and interquartile; SD=standard deviation. Statistical tests: t-test or Mann-Whitney test.
